# Refugees, asylum-seekers and undocumented migrants and the experience of parenthood: a synthesis of the qualitative literature

**DOI:** 10.1186/s12992-017-0299-4

**Published:** 2017-09-19

**Authors:** Lisa Merry, Sandra Pelaez, Nancy C. Edwards

**Affiliations:** 1 School of Nursing, University of Ottawa, Ottawa, Canada; 2 Faculty of Education, McGill University, Montreal, Canada

**Keywords:** Migration, Parenthood, Refugees, Asylum-seekers, Undocumented, Parenting, Mothers, Fathers, Parents, Transnationalism

## Abstract

**Objective:**

To synthesize the recent qualitative literature and identify the integrative themes describing the parenthood experiences of refugees, asylum-seekers and undocumented migrants.

**Methods:**

We searched seven online databases for the period January 2006 to February 2017. We included English and French published peer-reviewed articles and graduate-level dissertations, which qualitatively examined the parenthood experiences of refugees, asylum-seekers and undocumented migrants. We summarized study characteristics and performed a thematic analysis across the studies.

**Results:**

One hundred thirty eight studies met inclusion criteria. All but three were conducted in high-income countries, mainly in the US. Migrants studied were mostly undocumented from Latin America and refugees from Sub-Saharan Africa. Almost all studies (93%) included mothers; about half (47%) included fathers; very few (5%) included extended family members. We identified three integrative themes: 1) experiencing hardship and/or loss in the context of precarious migration and past traumas; 2) building resilience and strength by bridging language, norms and expectations; and 3) living transnationally: obligations, challenges and resources. Each theme contributed to shaping the parenthood experience; the transnationalism theme intersected with the themes on hardship and loss and resilience and strength.

**Conclusion:**

More research is needed with fathers, extended family members, asylum-seekers and in the LMIC context. A transnational lens needs to be applied to programs, policies and future research for refugee, asylum-seeker and undocumented migrant parents. Addressing transnational concerns (family separation and reunification), acknowledging transnational resources, fostering a transnational family identity and conducting transnational and longitudinal studies are potentially pivotal approaches for this sub-population of parents.

**Electronic supplementary material:**

The online version of this article (10.1186/s12992-017-0299-4) contains supplementary material, which is available to authorized users.

## Background

In many countries migrant families with children are a rapidly growing population, with migration for some, sparked by threats of war and violence, political and civil instability, and poverty [1–3]. Parenthood, whether for first or subsequent children, involves emotional, social and physical changes as well as adaptations of functional roles [4], which continue through childhood from infancy to teen years/young adulthood. While all families may face challenges, migrants may experience compounding difficulties due to the loss of social support networks including their extended family, adjustments necessitated by a new cultural context, experiences of discrimination, declines in social economic status, and reduced access to health and social services [5–8]. Refugees, asylum-seekers and migrants with undocumented status have particularly challenging migration trajectories. They may have suffered abuse and trauma pre- and during-migration and are more likely compared to other migrants, to be exposed to unfavorable and stressful conditions in the receiving-country, which puts these families at risk of marginalization [5, 8–12].

There is recognition that migrant families with children often face multiple difficulties and need specialized support [13–16], however less attention has been given to the unique parenthood contexts presented by having a humanitarian or precarious migration status [17, 18]. Greater understanding of the experiences of refugee, asylum-seeking and undocumented migrant families, especially from their perspective, could inform health and social services, practices and policies. Literature reviews are useful in this regard, especially when the literature is voluminous and complex. There is a large amount of primary research, predominantly qualitative studies, on the parenthood experiences of refugees, asylum-seekers and undocumented migrants and parenthood experiences [19–25], however no reviews of this body of literature were identified. The purpose of this review was therefore to synthesize the recent qualitative literature and to identify integrative themes describing the parenthood experiences of refugees, asylum-seekers and undocumented migrants.

## Methods

We used an integrative approach to our synthesis, which involved amalgamating and summarizing data from the qualitative literature [26]. Thematic analysis was used to identify integrative themes that reflected and described the parenthood experiences of refugees, asylum-seekers and undocumented migrants across the research [27–29].

### Search strategy

We searched seven online databases: EMBASE, Medline, Global Health, CINAHL, PsycINFO, sociological abstracts, and social work abstracts. The searches in CINAHL and EMBASE excluded Medline records. The search strategy was developed in consultation with a university librarian. Subject headings and keywords used related to parenthood (e.g., motherhood, fatherhood, parenting, child rearing) and migration (e.g., immigrant, refugee, immigration, asylum) and terms were adjusted depending on the standardized vocabulary used for each database. The searches for migration and parenthood were combined using the AND Boolean operator. Keywords were searched only within the titles, abstracts and keywords. Searches were limited to English and French literature and to the period of January 2006 to February 2017. An example of a detailed search strategy (i.e., MEDLINE) can be found in Table 1.

**Table 1:** Refugees, asylum-seekers and undocumented migrants and parenthood experiences: MEDLINE Search strategy

1. exp. Parents/
2. Parenting/ or Child Rearing/
3. (mother* or father* or child rearing or child birth or childbirth*).ti,ab,kw.
4. 1 or 2 or 3
5. exp. Human Migration/
6. “Emigrants and Immigrants”/
7. Refugees/
8. “Transients and Migrants”/
9. (immigra* or emigra* or refugee* or asylum or foreigner*).ti,ab,kw.
10. 5 or 6 or 7 or 8 or 9
11. 4 and 10
12. limit 11 to (english or french)
13. limit 12 to humans
14. limit 13 to yr. = “2006 -Current”
15. limit 14 to (case reports or classical article or clinical trial, all or comment or comparative study or controlled clinical trial or editorial or evaluation studies or government publications or introductory journal article or journal article or letter or meta analysis or multicenter study or observational study or published erratum or randomized controlled trial or “review” or “scientific integrity review” or systematic reviews or validation studies)

The asterisk (*) is a wildcard symbol and represents any group of characters, including no character. The wildcard expands the search to include variations (spelling, various endings) of the search term

### Inclusion and exclusion criteria

The inclusion and exclusion criteria are summarized in Table 2. Studies must have included refugees, asylum-seekers and/or undocumented migrants and examined the impact of migration on parenthood experiences. The following definitions were used to determine whether or not eligible migrants were included in studies. *Refugees* refers to migrants who fled their country to escape persecution for reasons of race, religion, nationality, membership of a particular social group or political opinion [30]; *asylum-seekers* are those who left their country and are seeking refugee status in another country and awaiting a response to this claim [30]; and *undocumented migrants* are individuals who migrated through irregular channels (i.e., movement outside of regulatory norms without the necessary authorization or documents required under immigration regulations) or who remained in a country without authorization or documents required under immigration regulations [30]. ‘Parenthood experience’ was defined as ‘the state of being a parent and the responsibilities involved’ [31]. It included the experience of becoming a parent and/or the experience of parenting (i.e., the process of supporting the physical, social, psychological and intellectual development) a child/children including teenagers and youth.

**Table 2:** Refugees, asylum-seekers and undocumented migrants and parenthood experiences: Inclusion and exclusion criteria

Inclusion Criteria
1. Included refugees, asylum-seekers and/or undocumented migrants^a^
2. Focused on the parenthood experience (i.e., experience of becoming a parent and/or raising and caring for children)^b^
3. Was empirical research (i.e., dissertations or published journal articles; excluded commentaries, theoretical/discussion papers, reviews, books, book reviews, editorials, conference abstracts/proceedings, or newspaper/magazine articles)
4. Qualitative or mixed methods research designs that had qualitative data reported as themes
5. Was written in English or French
6. Published between January 2006 to January 2016 (search updated to also include literature from period of January 2016 to February 2017 inclusive)
Exclusion Criteria
1. Examined the migration-parenthood experience only in the context of illness or disability (child or parent)
2. Described only the experience of transnational parenting (i.e., parents who migrated and were parenting from a distance, children who remained in the country of origin)^c^
3. Focused on parenting styles and (cultural) beliefs without any reference to how migration had an influence on parenting practices and beliefs
4. Described the behaviors, actions, experiences of migrant parents in a new country without any mention of the role of migration on these
5. Studied the migration-parenthood experience only from the perspective of a healthcare professional/service-provider and/or children. Or the text/media (data sources) were not produced by parents
6. Tested a parenting intervention
7. Described the development of an instrument

^a^ *Refugees* are those who fled their country for reasons of persecution; *Asylum*-*seekers* are those who are seeking refugee status in another country and awaiting a response; and *Undocumented migrant*s are those who migrated through irregular channels (i.e., movement outside of regulatory norms including entry and stay without the necessary authorization or documents required under immigration regulations). Studies may also have included other migrants in their samples

^b^ *Becoming a parent* included women’s experiences of pregnancy and giving birth only if it included the experience of becoming a mother. *Parenthood experiences* included the experiences of mothers, fathers and also other individuals parenting children (e.g., grandparents)

^c^Studies that described the experience of parenthood in the context of reunification (parents and children in the new country) or discussed issues of transnational parenting as it related to the parenthood experience in the new country, were included

The literature was restricted to empirical studies with qualitative or mixed methods research designs. The latter must have included a qualitative component. Methodological quality was not an exclusion criteria since the intention was to review and report on the literature broadly. Studies must have reported the perspectives of those experiencing parenthood including mothers, fathers and/or others (extended family members or guardians) involved in parenting a child in the settlement country (i.e., children had to be in the new country). Studies examining the parenthood experience in the context of illness or disability (parent or child), were excluded. The geographic location of the study could have been anywhere, including a high-, low- or middle-income country. We considered peer-reviewed articles as well as dissertations; if results from a dissertation were also duplicated in a published report, only the latter was retained. Published articles that had duplicate data were kept when additional and relevant data were reported in one paper but not the other(s) and/or the focus and analysis of the data were different.

We downloaded and managed all citations using Endnote X7 software. LM screened all titles and abstracts for eligibility. When eligibility could not be ascertained, the methods and results’ sections of the papers were reviewed. SP independently reviewed 10% of the titles/abstracts to determine consistency in the selection process. The rate of agreement between the two reviewers for eligibility was 92%. Discrepancies were resolved by fully perusing the papers and coming to a joint decision through discussion.

### Data extraction, collation and reporting

For all papers that met the inclusion criteria, we extracted and stored data in an excel database. Data extracted included: 1) paper characteristics [i.e., publication year, language, and discipline (based on the academic credentials and/or department of the first author or the journal if the first author’s information was not stated)]; and 2) study information, including the objective, research design, the data collection methods, country location and migrant population studied. The research design was based on what was stated in the article (i.e., mixed-methods, ethnography, qualitative descriptive, grounded theory, narrative inquiry, case study, phenomenology). In cases where it was not explicitly named or it was unclear, we categorized the study design based on the description provided. General ‘qualitative’ exploratory studies were categorized as ‘qualitative descriptive’ [32]. For mixed-methods research designs, the design for the qualitative component(s) was also documented. Data collection methods were categorized into: interviews (including semi-structured, informal, structured interviews), observation (participant or non-participant observation), focus groups and other (e.g., photovoice, journals, field-notes, questionnaires, text review). We also noted whether migrants’ receiving-country language ability was a criterion for study participation.

We categorized the country where the study was conducted as either a high-income country or a low-or middle-income country (LMIC) (based on the World-Bank Classification). For ‘migrant population’ we recorded the exact description as reported in the paper (e.g., Latinas) as well the migrants’ origins (categorized according to world regions), the migrant group (refugee, asylum-seeker, undocumented) and whether or not data on migrants’ length of time in receiving-country was collected and considered. We recorded whose (mother, father and/or other person such as a grandparent) parenthood experience was examined and the age-group of the children (e.g., school-age, teenagers). All paper and study characteristics were descriptively analyzed.

All of the original descriptors and descriptions of categories, themes, and sub-themes from the results’ sections of the studies that were relevant to the research question were also extracted and compiled into the excel database. The extracted data were entered into columns organized by whose perspective was reported- mothers, fathers, and mixed parents (mothers, fathers and extended family members). Details specific by migration status (refugee, asylum-seeker, undocumented) were noted within each column. For studies where results were reported for migrant (refugees, asylum-seekers, undocumented migrants and other migrants) and/or parent (mothers and fathers) groups together, extraction involved identifying and recording specific experiences within the primary studies’ results that were associated with the sub-groups.

The thematic analysis was led by LM. All extracted themes, sub-themes and categories and accompanying descriptions were reviewed and coded. A coding framework was developed iteratively by incorporating the content and essence of the original study themes into codes that represented the parenthood experiences from across the studies [26, 28]. Codes were compared by ‘parent group’ (mother vs. father) and migrant group (refugee vs. undocumented; there were too few studies to compare asylum-seekers) to see if there were particularities within, or identifiable patterns in experiences across these sub-groups [26, 33]. To address the limitation of combined results (migrant and/or parent groups), comparisons were also made between studies with combined results and those which reported on specific sub-groups (i.e., refugees, undocumented, mothers or fathers). The integrative themes were generated by organizing the codes into broader groupings and observing how they related to each other [29]. SP and NE reviewed the codes and the integrative themes and suggested refinements. Several discussions among all authors during the analysis phase were used to arrive at the final wording of the integrative themes. See Table 3 for an example of an integrative theme derived from the extracted original themes and sub-themes.

**Table 3:** Refugees, asylum-seekers and undocumented migrants and parenthood experiences: Example of derived integrative theme and original themes

Building resilience and strength by bridging language, norms and expectations	“Feeling fortunate” [159] “Fathers’ Self-Identified Coping Skills/Support System and Effects on Their Families” [52] “Coherence and hope: past, present and future” [53] “Coping with costs of getting ahead: familism, strict parenting, cultural traditions and rituals” [39] “Benefits of living between two cultures” [38] “Personal resources: faith and spirituality” [161] “Community networks” [161] “Managing Work-Care Reconciliation: Formal and Informal Resources” [56] “Standing for myself: self-supporting, creating new roles and identities” [75] “Self-responsibility and self-advocacy” [19] “Community” [19] “Spiritual foundation” [19] “Family” [19] “Access to language proficiency” [19] “Extended family as resources” [138] “Relationships as resources” [58] “Spirituality” [58] “Cultural maintenance” [58] “Secure states of mind: weaving coherence and continuity into a fragmented life history” [163] “Community building within and across ethnic boundaries” [78] “Benefits of coming to Canada” [47] “Coping strategies and resources: developing/drawing on skills, seeking healthcare, using internal strategies, living with emotional state, using relational strategies (formal and informal), drawing on other strategies” [70] “Coping resources: dispositional, health, skills, social, tangible resources; 3-processes and facilitators: social inclusion processes, facilitators (financial, social, other)” [70] “Interventions: daycare, education, employment, food support, health care, housing, immigration support, psychosocial care, organizations/programs (community supports), social groups activities, welfare/financial assistance” [70] “Countering micro-aggressions” [91] “Ordinary nature of resilience” [42] “Dynamic process of resilience in each and every day” [42] “Overcoming Barriers and Building Bridging Capital = agency and optimism” [116] “Transforming suffering into life lessons” [165] “Fomenting Courage” [165] “Adapting to immigration and new environment” [162] “Meaningful, purposeful and enjoyable leisure as a means to adapting to new life Challenges” [162] “Family benefits and opportunities in coming to Canada” [61] “Parental resources and strategies (family siblings, religion and support)” [43] “Closeness and communication with extended family members” [44] “Motherhood as a turning point” [81] “Augmenting joy: mother pulling herself together” [94] “The ways in which mothers adapted to the post-migration setting and found new ways of parenting” [146] “Perceptions of the supports available” [25] “A deep sense of gratitude to this country” [142] “Symbolic meaning attached to the use of Spanish” [142] “Resistance Against Patriarchy” [45] “Modeling cultural heritage and religious socialization” [139] “Group solidarity” [139] “Confronting discrimination” [139] “Familial survival tools from the past carried into the present” [84] “Transmitting cultural values” [144] “Family closeness, family support” [144] “Language maintenance” [144] “It’s Most Important That You Spend Time with Your Child” [145] “Blood Is Thicker Than Water” – “The Close Kinship in Vietnamese Culture” [145] “Negotiating a seat at the table” [117] “Congolese Parents’ Strategies to Overcome Stereotypes” [127] “Parenting: Transmitting values and preparing her children for life” [129] “Importance of community” [128] “Learning English, bilingualism and empowerment” [96] “Importance of maintaining traditional practices” [154] “Model advocacy and advise children to advocate for their themselves and others” [164] “Social agency and transmitting values to the next generations” [20] “Engaging with advocacy networks” [21] “Taking the good with the bad in family life” [22] “Successful Confrontations” [104] “Supporting the Formal Education of Loved Ones in Their Own Ways” [136] “Valuing Bilingualism, Biliteracy, and Bilingual Education” [136] “A Mother’s Agency: Mothers as Sources of Knowledge” [130] “Mothers as Information Seekers” [130] “Parenting Support and Service Provision Awareness” [158]

## Results

**Fig. 1:**
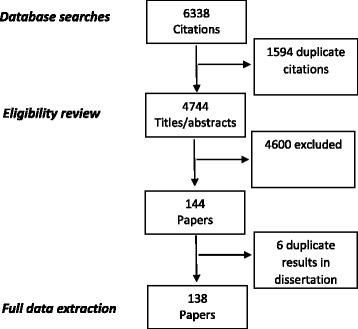
Refugees, asylum-seekers and undocumented migrants and parenthood experiences: Identification of literature

Figure 1 shows results from the database searches. The searches yielded 6338 citations and 4744 titles and abstracts after duplicates were removed. One-hundred and thirty-eight papers met the inclusion criteria for this integrative synthesis.

Table 4 summarizes the characteristics of included studies. Most of the research was published between 2011 and 2017. All papers were in English and the vast majority of studies were conducted from a social-sciences’ perspective. Almost all studies had been conducted in high-income countries, predominantly the United States (54%; *n* = 75), followed by European countries (15%; *n* = 20), Australia (14%; *n* = 19) and then Canada (12%; *n* = 17). Only three studies (2%) had been conducted in LMICs (Morocco [34], South Africa [35] and the Dominican Republic [36]).

**Table 4:** Refugees, asylum-seekers and undocumented migrants and parenthood experiences: Descriptive Summary of Literature

Descriptor	Studies *N* = 138, %(n)
Year of publication	
Jan 2006- Dec 2010	31.9% (44)
Jan 2011- Feb 2017	68.1% (94)
Discipline^a^	
Health Sciences	17.4% (24)
Social Sciences	82.6% (114)
Location of study	
United States	54.3% (75)
Europe^b^	14.5% (20)
Australia	13.8% (19)
Canada	12.3% (17)
New Zealand	0.7% (1)
Israel	2.2% (3)
Low- or middle-income country (LMIC)^c^	2.2% (3)
Migration Source Region^d^	
North Africa and/or Middle East	13.8% (19)
Sub-Saharan Africa	39.9% (55)
Caribbean	3.6% (5)
Latin America	44.9% (62)
Eastern Europe or Russia	8.7% (12)
South Asia	8.0% (11)
South East Asia	15.2% (21)
East Asia	0.7% (1)
Unspecified Asia	1.4% (2)
Migrant group^d^	
Refugee	54.3% (75)
Asylum-seeker	7.2% (10)
Undocumented	44.9% (62)
Parents	
Mothers	50.7% (70)
Fathers	6.5% (9)
Mothers and fathers	37.7% (52)
Mothers and extended family	2.2% (3)
Mothers, fathers and extended family	2.9% (4)
Child Age Group^d^	
Pregnancy	8.0% (11)
Infant and/or toddler	18.1% (25)
Pre-school	15.9% (22)
Primary School	29.0% (40)
Adolescent and/or Youth	30.4% (42)
“Childhood”	11.6% (16)
Adult Child	5.1% (7)
Unspecified ages	23.9% (33)

^a^Fields most represented were education (*n* = 27), social work (*n* = 26), psychology (*n* = 23), nursing (n = 10) and sociology (n = 9)

^b^Includes Belgium, Denmark, Finland, Northern Ireland, Italy, Netherlands, Norway, Sweden and the United Kingdom

^c^LMICs include: Morocco, South Africa, and the Dominican Republic

^d^A study may be counted in more than one category so percentages do not add to 100%

Migrant groups studied were mostly from Latin America, Sub Saharan Africa and South East Asia. Latin American migrants were primarily undocumented migrants living in the US (37% of studies, *n* = 51), while Sub-Saharan African and South East Asian (Vietnamese and/or Cambodian) migrants were mainly refugees (35% of studies, *n* = 48; 10% of studies, *n* = 14 respectively) living in a range of countries including Australia, the US, European countries and Canada. Only 7% (*n* = 10) of studies included asylum-seekers. One study focused on undocumented migrants who were sex-trafficked women who migrated to Israel from the former Soviet-Union [37]. The majority of studies (93%; *n* = 129) included mothers; in 54% (*n* = 70) of these participants were exclusively mothers. Approximately half of the studies included fathers (47%; *n* = 65); a small portion of these, 14% (*n* = 9), focused specifically on fathers. Only 5% (n = 7) of all studies included extended family members. Study populations were most often parents of school-aged children (29%; *n* = 40) and teenagers/youth (30%; *n* = 42).

Additional file 1 provides more detailed information about each individual study. Populations were mostly described only by country or region of origin. Just over one-fifth of studies (22%; *n* = 31) also described their population by ethnicity/religious identity (e.g., Latino, Hmong, Muslim); and fewer, 2% (n = 3) also used race (i.e., Black). The majority of studies (67%; *n* = 92) included other migrant groups (e.g., economic and family sponsored immigrants) in addition to refugee, asylum-seeking and undocumented migrants. Participants were ‘recent arrivals’ in 28% (*n* = 38) of studies, usually defined as less than five or 10 years; four studies (3%) purposely focused on more established migrants. Twenty-two percent (*n* = 30) did not provide any information about length of time since migration. In most studies with both mother and father participants (59%; *n* = 33 of 56 studies), the number of males was much smaller than the number of females.

A range of qualitative research designs were used including ethnography (27%; *n* = 37, 2 of these were focused ethnographies), qualitative descriptive (31%; *n* = 43, 2 of these used interpretive description), grounded theory (18%; *n* = 25), case study (10%; *n* = 14), narrative inquiry (7%; *n* = 10) and phenomenology (7%; n = 10). Five percent of studies (*n* = 7) were mixed methods research designs. Data were mostly gathered via interviews (86%; *n* = 119), observation (26%; *n* = 36) and/or focus groups (25%; *n* = 34). A handful of studies used other data collection methods such as photovoice, self-filming and journal diaries (11%; *n* = 15). Language of migrants was an exclusion criterion in 10% (*n* = 14) of studies. Thirteen papers (9%) overlapped in their data sources and reported duplicate findings [38–50].

The research primarily focused on migrants’ experiences since arrival in the receiving-country, reflecting on how their migration trajectory had had an impact on their experiences. There were a few exceptions. One study, although conducted in the US, focused on migrants’ experiences raising children in refugee camps in Africa [51]. Another study described the experience of mothers while living in a ‘transit-country’ [34]. A number of studies examined the parenthood experience broadly [20, 24, 25, 34, 36, 37, 41, 47, 48, 50–64], some were specific to the time around birth [65–70], and some discussed aspects related to identity and meanings of parenthood in the context of migration [22, 34, 36, 37, 47, 48, 61, 64, 67, 71–79]. Some studies focused on parenthood experiences under more challenging circumstances including single parenthood [23, 40–42, 58], teenage pregnancy [69, 80–83], having an undocumented or mixed status family [21, 84–96], reunification with children who migrated at a later time [97], and mothering in the context of conjugal violence [98–100].

Several studies examined specific parenting topics including: navigating the healthcare system [101–105] or other services [19, 23, 88, 92, 95]; decision-making associated with vaccinations [106], breastfeeding [107–109], nutrition [110–113] and oral health [114, 115]; interacting with schools and expectations regarding education [49, 89, 96, 104, 116–137]; maintaining traditions, beliefs, and languages [45, 74, 78, 84, 138–145]; raising teenagers [43, 44, 122, 124, 146–148]; and socializing and disciplining children, including involvement with child protection services [149–156]. Other studies had broader foci including: acculturation/bi-cultural development and parenting [20, 38, 44, 57, 58, 143, 144, 148, 157, 158]; the effect of migration on relationships between parents and children [19, 20, 38, 39, 60, 62, 147, 148, 159, 160], the extended family [44] and community [41]; strengths and coping to overcome parenting stressors [19, 42, 43, 52, 53, 70, 161, 162]; the effects of trauma on parenthood [22, 40, 51, 106, 159, 160, 163]; the negative impact of immigration and related policies on family life [25, 54, 87, 88, 90, 92, 93, 105, 164]; and dealing with discrimination as a parent [35, 46, 91, 164].

### Refugees, asylum-seekers and undocumented migrants and parenthood experiences: Integrative themes

**Fig. 2:**
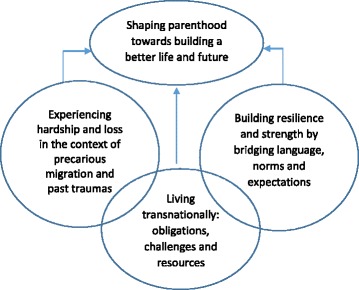
Refugees, asylum-seekers and undocumented migrants and parenthood experiences: Themes

We identified three integrative themes across the studies: 1) experiencing hardship and loss in the context of precarious migration and past traumas; 2) building resilience and strength by bridging language, norms and expectations; and 3) living transnationally: obligations, challenges and resources. Each theme contributed to shaping the parenthood experience; the transnationalism theme intersected with the themes on hardship and loss and resilience and strength. Themes are diagrammatically depicted in Fig. 2 and detailed descriptions (codes) are reported in Table 5.

**Table 5:** Refugees, asylum-seekers and undocumented migrants and parenthood experiences: Integrative themes and description

*Theme*	*Description (Codes)*
Experiencing hardship and loss in the context of precarious migration and/or past traumas	- Resettlement challenges/hardships affecting families include: under/unemployment or low wage, precarious and exploitive employment, poverty/low income, poor housing conditions, unsafe neighborhoods, and marginalization and discrimination due to social status, education level, race, gender, language and/or migration status - Consequences of resettlement challenges/hardships: less family time (survival becomes priority), reduced access, eligibility and barriers to services (e.g., healthcare, childcare, education) and resources (food, shelter), compromised parenting (unable to provide as needed), feelings of stress/overwhelmed, guilt and anxiety, and concern for children’s safety, well-being and upward mobility - Loss of family and community (no support and help to raise children, isolation and sadness, single parenthood) - Unfamiliar systems, language barriers, discrimination and different expectations (education, disciplining, child supervision, values, socialization, health) - Family and community tension and conflict due to changing roles, expectations and power dynamics - Overcoming and coping with past traumas (war, loss, rape) (difficult interaction, attachment, and communication and intensified concerns about safety and protection of children) - Living with fear and uncertainty: fear of family separation or being forced to return to country (deportation), fear of accessing services and restricting movement because of fear of deportation and/or fear it will affect future status
Building resilience and strength by bridging language, norms and expectations	- Family and community closeness as sources of strength and support - Faith, church and religion as source of strength and coping - Pride in nationality (to maintain identity and a positive sense of self in order to resist assimilation and oppression, overcome hardships and gain upward mobility) - Importance of maintaining and transmitting language, culture, values and religion to children (maintenance of identity, social and cultural capital, keeping family connected, protecting children/family, keeping children healthy, and educating children) - Children as sources of support for parents and family (to bridge language and culture gaps) - Altering notions and enactments of parenthood because of circumstances and/or adapting for integration purposes (family size, role, values and involvement of parents, nutrition and feeding) - Letting go of culture, language and religion (for integration purposes and also not to carryover negative tensions from country of origin) - Empowerment due to changing gender roles and dynamics - Learning new languages, skills and ways of doing (empowerment for women, economic capital, role model for children) - Wanting children to learn new languages and skills and to be exposed to diversity (social, cultural and economic capital) - Resettlement assistance and/or government and community programs helpful - Determination and agency (seeking information and resources, becoming a resident/citizen, enacting and fighting for rights, overcoming stereotypes) - Appreciation for safety and access to resources, services and education for children - Positive interactions with receiving-country population as source of support (increased sense of belonging, practical support, social capital) - Laws and policies that provide rights and access to services - Children’s future as reason for migration - Motivated to succeed, rebuild life and survive for children - Children (having a baby) as meaning and purpose - High (educational) expectations for children (towards better life)
Living transnationally: obligations, challenges and resources	- Leaving family, including children, and friends behind for a better life (sadness) - Hopes to bring children and/or other family members to new country in future - Distance parenting - Remittances and providing for family left behind - Concern and tension between providing for family and children left behind and family in new country - High (educational) expectations for children towards better life for family left behind, and broader community - Changing family composition (deportation or reunification of family members) and adjusting relationships - Maintaining connections with friends and family in home country as source of support (social support, parenting advice) - Maintaining connections with friends and family in home country as means to transmit values, language and culture to children - Maintaining connections to resources (employment, access to healthcare, schools) in country of origin - Developing and fostering in children a sense of transnational identity and sense of belonging (preparing children for potential to return to country)

#### Experiencing hardship and loss in the context of precarious migration and past traumas

Common and core to the migrant parenthood experience were sacrifice, hardship and loss. In studies with undocumented or mixed-status families and asylum-seekers, the uncertainty of their future in the receiving-country impacted many facets of their lives and was key to their hardships [21, 25, 49, 52, 54, 56, 60, 72, 74, 76, 81, 85, 87–95, 100, 103, 134, 141, 143, 148, 152, 157, 164, 165]. Those without status lived in fear of deportation and separation from children and family [21, 60, 72, 87–89, 91–94, 99, 141, 147, 152, 157]; parents were in the difficult position of having to discuss their precarious status with their children and to prepare for the outcome if they were ever deported [85, 90, 164]. Not having status restricted their movement and interactions [21, 49, 50, 54, 72, 87, 88, 90, 92, 93, 95, 99, 105, 133, 134, 141, 147, 165] and adversely impacted their safety (since migrants would not seek help, including for family violence) and had profound effects on their mental well-being [72, 94, 99, 100, 102]. Undocumented families were frequently ineligible for services and/or would not seek services fearing that this action might affect their future status [21, 56, 72, 87, 88, 92, 105, 151, 152, 157, 165].

Asylum-seeking parents also feared separation from their children if their applications for refugee status were not accepted. They worried about being returned to an unsafe country with their children [24, 25, 81, 82]. Access to services and rights, such as being allowed to work, varied depending on the receiving-country [24, 25, 68, 81]. Like undocumented families the precariousness and difficult circumstances caused strain and affected the well-being of the whole family [24, 25, 68, 81, 82].

In studies with refugee families, the main contributors to their sense of loss and to the adversity that they faced, were the forced nature of their migration and experiences of war, violence and the death of family members and friends [22, 24, 53, 55, 62, 66, 81, 82, 98, 106, 139, 146, 154–156, 159–161, 163]. Some refugee mothers, including unaccompanied minors, experienced parenthood as the result of rape [81, 82]. Refugee parents were affected by past memories and worried about family and friends who still remained in their country of origin or were missing; these intensified concerns they had for the safety of their children.

Migrant parents in almost all studies described resettlement challenges (financial, housing, employment, access to services) [19, 23–25, 38, 39, 42, 47–50, 52–54, 56, 60, 63, 65, 69, 70, 72, 73, 75, 76, 81–83, 88, 89, 91, 92, 99, 103, 108, 112, 114, 119, 125, 127–129, 133, 139, 149, 155, 158, 161], which left them feeling overwhelmed. Discrimination and exclusion, especially due to their migration status, were frequently described as issues [24, 25, 46, 50, 54, 55, 58, 60, 68, 73, 74, 76, 87, 88, 90–93, 96, 100, 119, 127, 143, 147, 149, 156, 157, 161, 164, 165]. In many studies living in poorer neighborhoods augmented parents’ concerns about the safety of their children [19, 53, 57, 62, 73, 119, 127, 128, 148, 149, 153, 157, 161]. Financial strain was also common and caused parents to be consumed by work in order to provide for the family [24, 39, 47, 48, 50, 58, 65, 66, 90–92, 121, 125, 127, 129, 137, 144]; undocumented parents were particularly at risk of exploitive and poor working conditions [49, 52, 56, 76, 89, 91–93, 129, 152, 157, 165]. Parents in several studies reported needing to prepare and support their children to deal with discrimination [22, 24, 25, 46, 54, 55, 59, 68, 74, 76, 78, 96, 106, 125, 127, 129, 133, 139, 147, 164, 165]. Inadequate resources, the loss of family support, and concentrating on just surviving were issues shared by many parents across studies and it meant they struggled to meet the basic needs for children and could not parent as they would like [19, 20, 22–25, 39, 47–50, 55–60, 62, 63, 65, 66, 68, 74, 81, 90, 92, 106–109, 121, 123, 125, 127, 129, 146, 147, 149, 150, 153–155, 157, 161, 165]. This contributed to parents feeling demoralized, inadequate, and humiliated.

In almost every study parents described experiences of having to manage different languages, traditions, beliefs and ways of doing, including navigating new ‘systems’ (e.g., education, healthcare, child welfare services) [20, 23, 24, 38, 47, 48, 50, 53, 54, 57, 60–66, 73, 75, 78, 89, 96, 101, 103, 104, 106–117, 119–121, 123, 125–128, 130, 132–135, 137, 139, 144–147, 149, 150, 152–154, 156–158, 161]. This presented challenges regarding the socialization and disciplining of children and interaction with educators [20, 53–55, 57, 59–63, 65, 116, 117, 121, 123, 125, 127, 128, 132–135, 137, 146, 149, 150, 153–155, 157, 158, 161]. In some studies parents expressed that they felt silenced; some were concerned that their efforts to parent and support their children, especially academically, were not recognized or valued [64, 91, 96, 104, 118–120, 123, 128, 135, 137, 140, 141, 156]. In several studies, parents sensed a loss of control and authority over children and also worried that children would lose their language and culture; the acculturation gap between parents and children caused tension [19, 20, 38, 39, 47, 48, 53–55, 57–60, 62, 63, 73, 75, 117, 119, 125, 138, 149–156, 158, 161]. Some parents felt that ‘the system’ (teachers, social workers, child protection services) was working against them and parents perceived that they needed to conform to expectations of the receiving-society and feared losing their children to child protection services if they did not [19, 47, 48, 54, 55, 57, 60, 62, 63, 73, 98, 123, 127, 145, 146, 149, 151–153, 155, 156].

Changing roles and relationships due to cultural/linguistic shifts and because of changes in family structures related to migration, was also an experience that emerged across studies [19–21, 23, 24, 39, 40, 42, 52, 55, 62, 75, 79, 93, 98, 106, 110, 125, 127, 130, 132, 141, 142, 145, 146, 148, 152–155, 157, 158]. Notably single parenthood among refugee mothers was sometimes due to death or missing spouses [23, 40–42, 155, 158]. Changes in gender roles (between parents and children, and between parents) was also a common experience [19, 20, 23, 24, 39, 42, 43, 47, 48, 50, 52, 55, 59, 62, 67, 75, 79, 98, 123, 125, 126, 128, 130, 144, 147, 148, 154, 155, 157, 158]. New roles and relationships sometimes caused tension within families and within the larger community [20, 23, 24, 40–42, 45, 54, 55, 62, 67, 75, 78, 79, 93, 98, 127, 128, 149, 155, 158]. A number of studies suggest that fathers struggled more with shift in gender roles and social expectations [20, 24, 39, 52, 55, 67, 75, 79, 90, 98, 141, 155]. In some cases it resulted in isolation and oppression of women, and/or conjugal violence [23, 54, 67, 75, 76, 98, 125, 155].

Overall families in many studies felt that life in the new country was not exactly how they imagined it would be. Resettlement difficulties, particularly for undocumented migrants, hindered families from advancing as they would have liked and parents reported disappointment and needing to adjust their expectations due to these challenges [22, 24, 77, 89, 90, 116, 127–129, 148, 149, 165]. In some studies parents expressed a need for more information regarding their rights, eligibility and availability of support and services [23, 54, 61, 105, 133, 150, 158]. In several studies they requested more information about how systems (education, welfare, social services, health, and child protection) worked and their related legal frameworks and cultural expectations about parenting [23, 25, 54, 55, 57, 61, 63, 100, 114, 115, 119, 121, 126, 132, 133, 146, 150, 151, 155–157].

#### Building resilience and strength by bridging language, norms and expectations

Despite the many challenges that migrant families faced, what came through across the research is that they also demonstrated strength and resilience and drew on a number of resources (internal and external) for support [22, 39, 42, 43, 52, 57, 70, 77, 82, 89, 91, 94, 104, 112, 129, 130, 137, 158, 161, 165]. In some studies, strength came from focusing on the positive and feeling grateful to be living in a safe place, especially for those who left countries with unrest or for those who had experienced war [21, 22, 42, 47, 48, 57, 66, 70, 72, 122, 129, 142, 158]. Hope for a better life and future was commonly described as a source of resiliency [19, 25, 53, 55, 75, 76, 80, 81, 96, 104, 116, 122, 124, 127, 129, 133, 136, 139, 143, 149, 158, 164] and fueled parents’ determination to rebuild their lives and succeed in the new country [19, 21, 22, 40, 47, 48, 53, 71, 76, 77, 81, 83, 89, 94, 116, 129, 143, 148, 149, 158]. In many studies parents also described their children as giving meaning to their lives and justification for their families’ sacrifice and hardship [21, 22, 39, 40, 50, 52, 55, 62, 65, 69, 71, 75–77, 80–83, 89, 94, 96, 116, 122, 124, 126, 127, 129, 135, 143, 147, 158, 165].

Faith, family and (ethnic/religious) community closeness and leisure time were described across studies as key sources of support; they provided distraction from life stresses, eased distress, gave hope and also offered practical aid (e.g., childcare, information, advice, financial), including information on services and how to navigate systems in the new country [19, 21–23, 25, 38, 39, 42–45, 47–50, 55, 57, 58, 62, 70, 76, 81, 85, 89, 91, 92, 94, 95, 108, 109, 112, 121, 122, 127, 129, 136–138, 146, 158, 161, 162, 165]. In a number of studies the receiving-community provided resources as well [23, 38, 39, 72, 89, 104, 123, 129, 131, 145, 151, 162]. Positive interactions with the community and institutions enhanced the migrant family’s sense of belonging, and were a source of social capital (connections for employment, navigating the system). Parents considered learning new languages and ways of doing (or “letting go” of old ways) as empowering for themselves and as important for their children’s futures [19, 20, 38, 42, 45, 46, 49, 55, 58, 61, 65, 67, 75–79, 84, 96, 116, 125, 127, 130, 136, 138, 139, 141, 143–145, 148, 157]. Education was viewed across studies as the primary means for children to have a better life in the new country and parents put a lot of emphasis on the importance of their children obtaining an education to optimize their employment prospects [24, 47, 48, 50, 52, 53, 60–63, 89, 96, 106, 116, 120, 122, 124, 126–131, 133, 135–139, 147, 149, 157]. Resettlement programs (health, education, child care, social programs) when accessible, were described as helpful and allowed families to get ahead [49, 55, 57, 61, 70, 75, 95, 104, 112, 129, 137, 138, 143, 146, 149, 157, 158, 162]. Generally, studies showed that refugees had more rights and fared better (compared to undocumented migrants) because they had better access to resettlement programs and services [50, 55, 57, 61, 70, 75, 146, 158, 162].

A key aspect to families’ resilience across studies was the maintenance of language, culture, values and religion. Transmitting language, culture, values and religion was not only described as a form of building capital (social, economic, cultural), keeping children safe (by being strict), and maintaining an identity for families [20, 24, 38, 42, 46, 49, 53, 55, 63, 70, 73, 74, 78, 84, 108, 111, 127, 139–142, 144, 155, 164], but also perceived as a source of pride, strength and coping [38, 43, 46, 55, 57, 70, 75, 78, 91, 116, 127, 139, 161, 164]. It was a way for families to maintain closeness and resist internalizing negative perceptions about their culture and status and downward mobility [20, 46, 73, 74, 91, 127, 139, 140, 143, 145, 164]. Families used a range of strategies to pass on languages and values, including visiting and maintaining contact with friends and family in their country of origin [20, 42, 43, 74, 117, 125, 127, 139, 140, 143–145].

#### Living transnationally: Obligations, challenges and resources

In many studies families described their parenthood experience as having a continuing and pervasive transnational dimension (i.e., links with their home country) [20–24, 44, 46, 49, 50, 53, 55, 56, 58, 68, 73–75, 81, 84, 87, 88, 90, 108, 112, 114, 116, 122, 125, 127, 129, 130, 135, 139, 140, 145, 146, 148, 161, 165]. Families remained connected to their home country in diverse ways; their lives were affected by these transnational relations and activities. These ties added a layer of complexity to their hardships and losses, but were also a source of strength and support that contributed to families’ resiliency.

Family separation, including parents’ separation from their children, was common across all classes of migrants and was a major source of concern in a number of studies [22–24, 53, 55, 58, 68, 75, 81, 84, 87, 135, 139, 145, 146, 161]. Migrant parents were supporting these family members by sending remittances and also parenting children who remained in their countries of origin [21, 24, 49, 50, 56, 58, 68, 84, 87, 112, 127, 129, 135, 145, 161]. In some studies parents reported feeling guilty and torn between their family/children living in the receiving-country and those back home [58, 87, 135, 161, 165]. Some refugee and asylum-seeking parents felt particularly worried about the safety of their children and family  who remained in their home country; in some cases they  did not even know where their family members were or if they were alive [22, 23, 116]. Undocumented parents in some studies struggled with not being able to travel back to visit their home country for fear they would be caught by authorities and be unable to return to the receiving-country [84, 88, 90, 140, 148, 165].

Transnational ties were also maintained through serial migration and deportation of family members. In some studies families and parents spent years separated before spouses, children and/or other family members would join them [24, 56, 68, 135, 143]. In other instances, families lived with the hope of eventual migration of family members but were uncertain if it would ever be realized because of their precarious migration status or because of the administrative and financial challenges encountered in sponsoring family members [23, 24, 56, 68, 135]. For undocumented migrants, deportation was a constant threat and in the few studies where it had occurred and it was discussed, fathers were the ones who were targeted and returned to their home country [90, 93, 141]. The impact of serial migration and deportation led to families being fragmented and dealing with issues such as single parenthood and reunification [21, 39, 55, 68, 93, 97, 135, 141]. This included altered support systems, changing family dynamics and relationships, and/or added responsibility and stress, and community stigma (i.e., being a single mother) [39, 58, 68, 87, 135, 141]. Where reunification with family, particularly children, had taken place, parents described the challenges of learning to live with each other again and coping with emotions of having been separated (e.g., parents felt guilty, children felt resentment) for lengthy periods of time [39, 55, 58, 87, 97, 121, 135].

Transnational ties were also used as a positive resource. Parents in many studies drew on support from family and friends as a source of social support and parenting advice for their children [22, 44, 84, 108, 114, 127, 130]. Maintaining family connections in the home country, including visiting and sending children to visit, was described in many studies as a means to maintain their culture and traditions and to teach children their language [20, 44, 74, 84, 122, 125, 130, 135, 140, 145]. A few studies reported that parents also continued to maintain active involvement (e.g., work, return to use health services) in their countries of origin which enhanced resources available to the migrant family [46, 127, 130, 140]. Families in some studies envisioned returning to their country in the future, and some were also preparing for the eventuality that their children would go live and work in their home countries as adults [38, 62, 84, 127, 139]. Similarly, although less positive, some undocumented families were also preparing for the possibility of deportation [21, 85, 141, 157]. Lastly, maintenance of connections was described in some studies as serving as a source of resilience by preserving their identity and positive sense of self, which were essential in coping with loss and resisting oppression [46, 73, 74, 84, 116, 122, 140].

## Discussion

There is an extensive amount of qualitative research on the parenthood experiences of refugees, asylum-seekers and undocumented migrants; this is the first review, however, to synthesize and identify integrative themes from this body of literature. The themes regarding hardship and loss, and building resilience and strength*,* are consistent with observations made by others regarding the migration experiences of refugees, asylum-seekers and undocumented migrants generally [11, 166, 167], while the third theme on transnationalism highlights additional obligations, challenges and resources that need to be better understood and considered by care and service-providers working with these families. This theme also suggests a lens through which policies and research may be approached.

In the context of parenthood, our review shows that migration and resettlement stresses compound the responsibilities and concerns related to raising and caring for children in a new country and may have deleterious effects on the family [17, 54, 73, 98, 168]. Many experiences identified are common across all types of migrants [15], however results highlighted distinctive challenges related to having a precarious and/or humanitarian status. Furthermore refugees, asylum-seekers and undocumented migrants mostly migrate from LMICs and the cultural, social and religious differences between these countries and high-income countries are great and exacerbate resettlement difficulties, further adding to families’ stress. Despite these additional hardships and losses, the literature also showed that these sub-groups are resilient. Similar to other research on migrants and resilience, family and community support, maintenance of language, religion and culture as well learning new languages and ways of doing, fueled resilience [169]. For refugee, asylum-seeker and undocumented migrant parents, children were also an important source of strength and motivated parents to overcome their difficult circumstances. Together these results reinforce that greater attention is needed to address the unique challenges that refugee, asylum-seeking and undocumented families encounter and that efforts towards supporting families should concentrate on approaches that enhance resilience and strength, especially by bridging language, norms and expectations.

Transnationalism as it relates to migration and families has been a long time focus in the fields of sociology and anthropology [170, 171] while in the health disciplines it has largely been absent. A “transnational perspective” acknowledges that migrants’ experiences extend across countries and that families are affected by their relationships that they maintain with family and friends, their continued economic and political involvement, and their ethnic and cultural attachment to the home country [172]. Work in this area has mainly focused on transnational mothering (parenting children who remain in the home country from a distance) [173, 174], impacts of migration on children and elderly who remain in the home country [175, 176] and economic effects for families and societies via remittances [177]. Little to no research however has directly considered how transnational ties impact parenthood with children in the new country. Our findings build on the research on parenthood and transnationalism [178] and suggest a transnationalism lens is relevant in the healthcare field. Similar to studies on transnational parenting, our results highlight the strong sense of obligation, stress and distress parents experience in parenting from a distance their children who remain in the home country [178]. In addition, our results show how transnational obligations, as well as stresses associated with serial migration and deportation of family members, affect family dynamics and relationships and how parents experience parenthood in the new country. Findings also show how transnational ties may be positive and provide resources and support for migrant families with children. Parents’ health and well-being are therefore inextricably linked to their transnational realities and a receiving-country-centric approach is limited and inadequate for understanding their experiences and addressing their needs. In order to develop more relevant policies and practices, including in healthcare, a transnational lens is needed [171, 179, 180].

Traditional parenting and family support programs tend to emphasize integration and center on resettlement concerns [181, 182]. Using a transnational lens in program and policy development and design would include fostering a transnational identity and sense of belonging. This may be achieved by supporting families to practice their traditions and faiths, and speak and learn (for children) their languages. It would also include encouraging families to maintain their networks and ties to their home country [116, 133]. This would include acknowledging and addressing transnational parenting concerns (supporting family members back home, parenting from a distance, family reunification, deportation of family members) [183], and also recognizing and valuing transnational ties as a resource for families that can be used to foster resilience by building cultural, social and economic capital for parents and children. A transnational approach also involves building trust and resolving cultural frictions by “bridging languages, cultures and norms” between migrant and non-migrant communities. This would consist of creating opportunities for migrants to share their culture and traditions with the receiving-country population [73, 133, 142] as well as ongoing efforts to allow migrants to learn new languages and become more familiar with the way of life in the new country [132]. For the receiving-community it would involve raising their awareness of migrant issues, enhancing their empathy and sense of responsibility for migrants and their families living abroad, and addressing negative discourses [184].

### Limitations and strengths

This review has a number of limitations. Firstly, two thirds of the studies included other migrant groups in their sample, and none of these studies reported results separately making it sometimes difficult to ascertain to what extent results reflected the experiences of refugees, asylum-seekers and undocumented migrants. Asylum-seekers were also under-represented in the primary studies, providing less insight about the experiences of these families. Similarly, very few studies included extended family members, and although close to half of the studies included fathers, there were proportionately fewer fathers than mothers in the study populations. Furthermore, in studies with both mothers and fathers results were always combined and reported together and there were no comparisons made by parent sub-group.

Secondly, we did not include other migrants groups, such as temporary agricultural or domestic workers, or mail-order brides or other migrants who may also have migrated under difficult circumstances and face similar challenges as refugees, asylum-seekers and undocumented migrants. They may have had some different perspectives due to the nature of their migration trajectory and status in the new country. Thirdly, given there were very few studies conducted in LMICs conclusions cannot be drawn regarding the parenthood experiences of refugees, asylum-seekers and undocumented migrants specifically in a LMIC context. The challenges, concerns, hopes and expectations for these parents are likely to be very different, especially for those living in refugee camps or for those transiting through during their migration to another country. Lastly, it is possible that we did not include some relevant literature, particularly studies conducted in LMICs, due to the language restrictions and because we did not include grey literature.

This review, however, does have many strengths. Our approach was inclusive; the literature covered a broad range of parenthood experiences and diverse populations living in a number of countries. The analysis was integrative and reflected a large body of research. Included studies had a number of methodological strong points. Language was infrequently an exclusion criteria (10%) and in most studies researchers accommodated diverse languages with bilingual research assistants or interpreters; populations were defined mostly using recommended indicators (country of birth, length of time in receiving-country, migration status) [185]; aims and objectives were clearly stated in all; and many studies presented a reflexive approach [186].

### Practice and policy implications

The literature touched on a range of parenthood topics and suggest implications for policies and practices across multiple sectors, including the social, health, educational, as well as the political spheres. These include development of policies that address the undocumented and mixed status situation of families [54, 70]; removing eligibility barriers for health and social services, particularly for undocumented migrants [25, 54, 70]; enhancing mental health services and support for migrants who have suffered trauma, including young mothers who have experienced pregnancy as the result of rape; improving communication regarding laws, rights and access to services to migrants [54, 150]; and better mechanisms in the community and schools to support parents with parenting including discipline and supporting their children in school [55, 150]. A transnational perspective implies sensitivity training of educators, healthcare-providers, social workers, and child-protection workers is needed regarding the effects of the migration trajectory, migration status in the new country and ongoing transnational ties, on migrants’ health and well-being [23, 55, 119, 162]. In practice it involves validating families’ transnational experiences and enabling families to cope more effectively by tapping into their strengths and resources, including their transnational resources to help families overcome and recover from their adversity [183, 187]. At the policy level, policies which promote cultural cohesion between communities and address family separation, are also required.

### Future research

We need to know more about asylum-seeker families, particularly how they navigate parenthood in uncertainty while they await a response to their asylum claim and also when they receive a negative response to their claim. Further inquiry on the experiences of fathers and extended family members is warranted as well [188]. The role of extended family is an important gap to address since in many cultures parenting is shared beyond the mother and father and research has shown they have an influence on families in the migration context [189, 190]. Extended family members may also have different experiences [138, 142]. Moreover, they can be a significant resource for families [44, 138]. Lastly, given that LMICs are host to the vast majority of refugees, and also receive significant numbers of asylum-seekers and undocumented migrants [191], research in these countries is needed as well.

Research on the parenthood experiences of refugees, asylum-seekers and undocumented migrants must also consider the transnational realities of these populations. This research should include the perspectives of family members in the countries of origin [188]. Longitudinal work that follows families over their migration trajectory (across countries), may also offer further insights on the effects of migration on parenthood experiences over time [188, 192]. Research should aim to further understand the impact of transnational ties on parenthood, including transnational obligations (parenting other children and family who remain in the home country) and the effects of serial migration and deportation of family members. Better knowledge of transnational resources, and how to optimize these could also inform interventions and services towards supporting migrant families with children. Examining whether current health and social interventions consider the transnational challenges of migrant families’ lives would be relevant as well. Healthcare providers and others working with migrant families (social services, schools), may then be better equipped to promote protective factors, mobilize untapped resources and support migrant families in dealing with their day-to-day life challenges.

## Conclusion

We synthesized a large body of literature and our integrative analysis highlighted differences in parenthood experiences by migration status (refugee vs. undocumented) and also by parent group (mothers vs. fathers). Results from the review suggest that to further understand the experiences of refugee, asylum-seeker and undocumented migrant families, and to better address their needs and enhance resiliency, a transnational lens is needed.

## Additional file

Additional file 1: Refugees, asylum-seekers and undocumented migrants and parenthood experiences: Summary of Literature (DOCX 65 kb)
