# Supplementary material for: Refugees, asylum-seekers and undocumented migrants and the experience of parenthood: a synthesis of the qualitative literature
Source: Global Health. 2017 Sep 19;13:75. doi: 10.1186/s12992-017-0299-4 (PMC5606020; doi:10.1186/s12992-017-0299-4)
Supplement: Additional file 1: — Refugees, asylum-seekers and undocumented migrants and parenthood experiences: Summary of Literature (DOCX 65 kb) [file 12992_2017_299_MOESM1_ESM.docx]

**Additional file 1: Refugees, asylum-seekers and undocumented migrants and parenthood experiences: Summary of Literature**

| **1^st^ Author year** | **Objective** | **Methodology^[[1]](#footnote-1)^** | **Country Location** | **Migrant parent description,**  **Migrant group^[[2]](#footnote-2)^** | **Sample^[[3]](#footnote-3)^,**  **Data collection methods^[[4]](#footnote-4)^** |
| --- | --- | --- | --- | --- | --- |
| Abrego  2011 | To show that the implementation of contemporary U.S. immigration laws are a form of legal violence. | Ethnography | United States | - Latino  - Undocumented | - 25 mothers from one study and mothers NR^[[5]](#footnote-5)^ from a second study^[[6]](#footnote-6)^  - Interviews and observation |
| Alvarado  2009 | To explore how Latino immigrant men self-identify stress and coping strategies, and the subsequent effects on their parenting. | Grounded theory | United States | - Latino  - Undocumented | - 13 fathers  - Interviews |
| Anderson 2014 | To examine how the migration experience influences newcomer mothers’ young child feeding and care practices and their children’s overall health. | Ethnography | Canada | - Sri Lankan Tamil and Latin American  - Asylum-seeker | - 32 mothers  - Interviews |
| Andrews  2013 | To document health beliefs and treatment decision-making and impact of migration histories and acculturative forces on health belief systems and therapeutic decision-making. | Ethnography | United States | - Hispanic  - Undocumented | - 36 families, mostly (92%) were mothers  - Interviews |
| Atwell  2009 | To explore the factors which impact upon parents' ability to envisage their children's futures and support them in setting and achieving their ambitions. | Ethnography | Australia | - Sudanese, Burmese, Afghan, Bosnian, and Liberian  - Refugee | - 4 mothers, 3 fathers, 3 guardians (sister, uncle, cousin)  - interviews |
| Ayón  2010 | To understand how Mexican parents’ perceive their voices (their concerns, dissatisfaction, and opinions) as integrated in child welfare cases and what factors hinder or promote this process. | Grounded theory | United States | - Mexican  - Undocumented | - 16 mothers, 3 fathers  - Interviews |
| Ayón  2014 | To learn what services immigrant families need to promote their families’ well-being within a context of stringent anti-immigrant legislation. | Grounded theory | United States | - Latino  - Undocumented | - 45 mothers, 7 fathers  - Focus groups |
| Ayón  2016 | To learn from parents about the  effects of anti-immigrant policies on families, and specifically the effects on their own and children’s experiences with discrimination. | Grounded theory | United  States | - Latino  - Undocumented | - 43 mothers, 11 fathers  - Interviews |
| Bacallao  2007 | To explore how immigration affected family relationships. | Grounded theory | United States | - Mexican  - Undocumented | - 7 mothers, 7 fathers  - Interviews |
| Bacallao  2009 | To assess how families balance different cultural influences in the acculturation process and to gain understanding on bicultural development. | Grounded theory | United States | - Mexican  - Undocumented | - 7 mothers, 7 fathers  - Interviews |
| Baergen  2007 | To explore the familial effects and consequences of mother tongue loss. | Narrative inquiry | Canada | - Spanish-speaking from Colombia, El Salvador, Guatemala and Central America  - Asylum-seeker | - 6 mothers and  grandmothers  - Interviews |
| Belliveau  2011 | To examine, using an "intersectional lens" (i.e., multiple marginalized locations), how mothers access/don't access resources for their children. | Grounded theory | United States | - Mexican  - Undocumented | - 20 mothers  - Interviews |
| Bergnehr  2016 | To describe how women depict their mothering strategies, and future aspirations for themselves and their children and in what ways welfare dependence is connected to their mothering. | Qualitative descriptive | Sweden | - Iraqi  - Refugee | - 16 mothers  - Focus groups |
| Betancourt 2015 | To identify and examine strengths and resources to overcome resettlement and acculturative stressors. | Grounded theory | United States | - Somali  - Refugee | - 23 mothers, 9 fathers  - Focus groups |
| Betts  2011 | To investigate how Somali women perceive their gender and sexual identity in a Canadian context. | Ethnography | Canada | - Somali  - Refugee | - 13 women  - Interviews and one focus group |
| Biggs  2013 | To examine how immigration experiences influence early childbearing. | Qualitative descriptive | United States | - Latino  - Undocumented | - 14 teen mothers  - Interviews |
| Bonizzoni 2014 | To provide an exploration of the work–family reconciliation processes of immigrant working mothers. | Qualitative descriptive | Italy | - Latin American and Eastern European  - Undocumented | - 56 mothers  - Interviews |
| Bonizzoni 2015 | To analyze changes mothers and children experience in their relationship due to separations and reunions. | Narrative inquiry | Italy | - Migrants from Latin America, Eastern Europe, and Philippines  - Undocumented | - 40 mothers  - Interviews |
| Bowie  2016 | To gain an understanding of first-generation Somali families’ experiences of parenting in the United States and to discover potential barriers to effective parenting with the goal to design supportive interventions. | Phenomenology | United States | - Somali  - Refugee | - 20 families (mothers and fathers)  - Interviews |
| Brownfield Baird  2009 | To investigate influences to health and well- being of refugee women resettled with their children. | Ethnography | United States | - Sudanese  - Refugee | - 10 mothers  - Interviews and observation |
| Burke  2015 | To understand the decision-making and concerns about HPV vaccination of mothers who experienced genocide and came as refugees. | Qualitative descriptive | United States | - Khmer (Cambodia)  - Refugee | - 25 mothers  - Interviews |
| Busch Nsonwu 2013 | To describe refugees’ strengths and challenges to family relationships and to describe refugees’ strengths and challenges as they engage with public services to achieve self-sufficiency. | Qualitative descriptive | United States | - Afghan, Bosnian, Colombian, Congolese, Cuban, Ethiopian, Iranian, Liberian, Serbian, Somali, Sudanese and Vietnamese  - Asylum-seeker and Refugee | - 28 families (more female than male participants)  - Interviews |
| Carolan  2010 | To explore the experiences and concerns of an African-born sample of pregnant women. | Qualitative descriptive | Australia | - African  - Refugee | - 18 women  - Interviews |
| Carpenter-Aeby  2014 | To examine one family’s attempts to preserve the oral history of the Hmong culture and to promote understanding across generations and community members from different cultural backgrounds. | Case study | United States | - Hmong (Thailand)  - Refugee | -1 family (mother and father)  - Interviews |
| Carranza  2007 | To focus on the strategies that mothers and daughters utilized to resist prejudice and racism in their settlement country. | Grounded theory | Canada | - Salvadorian  - Refugee | - 16 mothers  - Interviews |
| Carranza  2013 | To examine which values Salvadorian mothers and their daughters considered important to maintain or modify. | Grounded theory | Canada | - Salvadorian  - Refugee | - 16 mothers  - Interviews |
| Clark  2007 | To describe mothers with varying acculturation levels, their expectations and experiences with children’s health care services. | Focused ethnography | United States | - Mexican  - Undocumented | - 28 mothers  - Interviews |
| Clarke  2010 | To examine the adjustments and acculturation process of single Liberian mothers. | Phenomenology | United States | - Liberian  - Refugee | - 10 mothers  - Interviews |
| Cook  2016 | To discuss parents’ parenting strategies and their struggles to redefine and negotiate roles, freedoms and gendered expectations. | Qualitative descriptive | United Kingdom | - African (Zimbabwe, Kenya, Somalia, Sudan)  - Refugee | - 13 mothers, 7 fathers  - Interviews and focus groups |
| Crush  2014 | To show and discuss the levels and types of discrimination experienced by Zimbabwean migrant parents in South African schools. | Qualitative descriptive | South Africa | - Zimbabwean  - Asylum-seeker, Refugee and Undocumented | - 198 women and men (parents and guardians, slightly more women than men)  - Interviews and focus groups |
| Dabbagh  2014 | To explore the maternal socialization goals and practices designed to preserve their culture of origin. | Qualitative descriptive | Israel | - Lebanese  - Refugee | - 12 mothers  - Interviews |
| Davies Dewitt  2007 | To examine the self and social identity through narratives of reproduction. | Ethnography | Canada | - Cambodian  - Refugee | - 9 women  - Interviews and observation |
| De Haene 2010 | To explore trauma experiences, parental states of mind and attachment. | Case study | Belgium | - Migrants from Iraq, Kosovo, Pakistan, Russian Federation (Ingushetia), Somalia, Sri Lanka, and Sudan  - Refugee | - 7 mothers, 4 fathers  - Interviews |
| De Los Rios 2008 | To examine migration experiences and gender relations, ideologies, and identities of working class migrants. | Ethnography | United States | - Migrants from Mexico, El Salvador, Guatemala, Honduras, and Colombia  - Undocumented | - 20 mothers  - Interviews, observation and focus groups |
| Deng  2013 | To investigate the parenting issues and concerns that South Sudanese experience while resettling in New Zealand. | Qualitative descriptive | New Zealand | - South Sudanese  - Refugee | - 4 mothers, 2 fathers  - Interviews |
| de Ruiz  2010 | To explore the relationship between Mexican American mothers and their daughters as they negotiated a bicultural identity within their cultural contexts. | Grounded theory | United States | - Mexican  - Undocumented | - 7 mothers  - Interviews, observation and text review of relevant community literature |
| Doering-White  2016 | To illustrate how undocumented mothers describe their experience negotiating the aftermath and threat of deportation. | Qualitative  descriptive | United  States | - Latina  - Undocumented | - 7 mothers  - Interviews |
| Doucet  2011 | To examine the tactics that Haitian immigrant parents used to negotiate the boundaries around home and school. | Qualitative descriptive | United States | - Haitian  - Undocumented | - 34 mothers, 15 fathers  - Interviews and field- notes |
| Dumbrill  2009 | To enable refugee parents provide information (perspectives on parenting and their views and experiences of Canadian child welfare services) that might help child protection workers and agencies to better engage and work with refugee communities. | Qualitative descriptive | Canada | - Migrants from West Africa, South West and Central Asia  - Refugee | - 9 women and 2 men  - Photovoice, focus group and individual discussions |
| Earner  2007 | To hear immigrant parents describe their experiences with child welfare services; to identify barriers to services these parents encountered; and to advocate for changes in policy, program, and practice so that public child welfare services can effectively address the special needs of immigrant families, children and youth. | Qualitative descriptive | United States | - Migrants from China (Mainland), Mexico, Guatemala, and Ecuador  - Undocumented | - 9 mothers, 2 fathers  - Focus groups |
| Enriquez  2015 | To explore how immigration laws affect undocumented parents and their citizen children. | Grounded theory | United States | - 1.5 generation Mexican, and Guatemalan  - Undocumented | - 23 women and 9 men  - Interviews |
| Erel  2013 | To discuss identities related to ethnicity, nation, culture and citizenship and to show how migrant mothers’ cultural work is also an active engagement with cultural citizenship. | Case study | United Kingdom | - Kurdish  - Refugee | - 9 mothers  - Interviews |
| Este  2009  Sex Roles | To examine the perceptions and experiences as fathers, including meaning of fatherhood, the values that guide their behavior as fathers, their interactions with and aspirations for their children, and the challenges these men encounter as fathers. | Qualitative descriptive | Canada | - Sudanese  - Refugee | - 20 men  - Interviews |
| Este  2009  The Annals of the American Academy of Political and Social Science | To examine the perceptions and experiences of Russian immigrant and refugee Sudanese men as fathers. | Qualitative descriptive | Canada | - Russian and Sudanese  - Refugee | - 34 men  - Interviews |
| Feliciano  2011 | To understand infant-feeding choices among Hmong American women. | Qualitative descriptive | United States | - Hmong  - Refugee | - 13 mothers  - Interviews and questionnaires |
| Felter  2009 | To examine the education goals parents have for their adolescents. | Grounded theory | United States | - Indian and Salvadorian  - Refugee | - 30 mothers, 10 fathers  - Interviews |
| Fraser  2012 | To gain understanding of the experiences of single Black mothers’ mothering and to learn about how they construct masculinity and manhood and what they think about the fatherlessness of their sons. | Grounded theory | Canada | - Black Caribbean  - Undocumented | - 3 mothers  - Interviews |
| Fuster  2013 | To examine Mexican mothers' beliefs on social and moral development in light of their adaptation to the United States. | Case study | United States | - Mexican  - Undocumented | - 5 mothers  - Interviews, observation, and Q sort activity |
| Gagnon  2013 | To learn about what processes are used by migrant women to respond to maternal-child health and psychosocial concerns during the early months and years after birth and which of these enhance or impede their resiliency; and to identify population interventions which they feel best respond to these concerns. | Focused ethnography | Canada | - Migrants from Burundi, Columbia, Ghana, Guyana, Iraq, Mexico, Nigeria, Rwanda, Sri Lanka, St. Vincent, and Yugoslavia  - Asylum-seeker and Refugee | - 16 mothers  - Interviews and observation |
| Gallo  2013 | To investigate naturally occurring interactions (language) in homes and school to reveal how Mexican immigrant fathers’ participation shapes, and is shaped by their young children’s schooling. | Ethnography | United States | - Mexican  - Undocumented | - 7 fathers  - Interviews, observation, and self-filming |
| Ginocchio  2014 | To examine how parents understand educational opportunities and how they navigate and interact with education system. | Case study | United States | - Latinos (Mexico)  - Undocumented | - 4 mothers, 3 fathers  - Observation, ‘action meetings’, photobook and research journal |
| Groleau  2006 | To identify cultural factors involved in the abandonment of breastfeeding amongst Vietnamese immigrant women in Canada. | Narrative inquiry | Canada | - Vietnamese  - Refugee | - 19 mothers  - Interviews |
| Jaysane-Darr  2016 | To address the question how a community reproduces in a diaspora situation tied in with concerns about citizenship, ethnicity, and the nation. | Ethnography | United States | - South Sudanese  - Refugee | - 34 women and 21 men  - Interviews, observation, and participation in public events |
| Jessri  2013 | To explore from mothers’ perspective, the experience of breastfeeding and their perceptions of attributes of the health care system, community and society on their feeding decisions after migration. | Ethnography | Canada | - Middle Eastern (Iraq, Iran, Kuwait and Saudi Arabia)  - Refugee | - 22 mothers  - Focus groups and survey |
| Jimenez-Castellanos 2012 | To gain insight into the impact of micro-aggressions on the engagement of undocumented Latino immigrant fathers with their children. | Ethnography | United States | - Latino  - Undocumented | - 29 fathers  - Interviews and observation |
| Jonsdottir 2012 | To examine and compare educational aspirations of parents of 3 groups: Kurds in Iraq, Kurds in Norway and native Norwegians. | Mixed methods  Qualitative descriptive | Norway | - Kurdish (Iraq/Kurdistan)  - Refugee | - 4-5 parents (number of mothers and fathers NR)  - Interviews |
| Kanini Githemebe 2010 | To examine involvement (barriers and strategies) of African refugee parents in the education of their elementary school children. | Mixed methods  Qualitative descriptive | United States | - African (Burundi, Somalia, Congo, Sudan, Tanzania, Sierra Leone, Liberia, and Rwanda)  - Refugee | - 6 parents (number of mothers and fathers NR)  - Interviews |
| Kelly  2009 | To understand the decision-making processes of battered Latino women in situations involving intimate partner violence (IPV). | Qualitative descriptive | United States | - Latino  - Undocumented | - 17 mothers  - Interview and focus groups |
| Kelly  2016 | To explore how participants made sense of, and created meaning around parenting and family life in the UK. | Phenomenology | United Kingdom | - African and Middle-Eastern  - Refugee | - 6 mothers  - Interviews |
| Kennedy Cuero  2012 | To examine how Latinas’ participation in schools is affected by ideological messages that subordinately position them in terms of their ethnicity, class, and immigrant status. | Case study | United States | - Latino  - Undocumented | - 3 mothers  - Interviews and observation |
| Kimondo  2014 | To further understanding of how displacement by war affects mothers’ beliefs about childhood, and the social and cognitive competencies they desired and inculcated in their children raised in a refugee camp. | Phenomenology | United States | - Liberian  - Refugee | - 4 mothers  - Interviews |
| Kyriakakis 2010 | To ascertain the structural and cultural factors at play in the manifestation of abuse and help seeking. | Grounded theory | United States | - Mexican  - Undocumented | - 29 women  - Interviews |
| Lai  2009 | To examine the relationship between fathers' exposure to trauma and communication with adolescents. | Mixed methods  Qualitative descriptive | United States | - Vietnamese  - Refugee | - 16 fathers  - Interviews |
| Leidy  2010 | To explore barriers to positive parenting and family cohesion in Latino immigrants. | Qualitative descriptive | United States | - Latino (mostly Mexico)  - Undocumented | - 12 mothers  - Focus groups |
| Lenette  2013 | To argue that it is in the dynamic space of everyday life-worlds of refugee women that a more complex set of possibilities become enacted, which gives meaning to the processes rather than the traits of resilience. | Ethnography | Australia | - Migrants from Sudan, Democratic Republic of Congo and Burundi  - Refugee | - 4 mothers  - Interviews, observation, photovoice, and digital storytelling |
| Lenette  2014 | To explore meanings attached to widowhood and experiences of refugee resettlement. | Ethnography | Australia | - Sudanese  - Refugee | - 2 mothers  - Interviews, observation, photovoice, and digital storytelling |
| Lenette  2015 | To outline how mistrust can create difficult resettlement circumstances. | Ethnography | Australia | - Migrants from Sudan, Democratic Republic of Congo and Burundi  - Refugee | - 4 mothers  - Interviews, observation, photovoice, and digital storytelling |
| Levi  2014 | To explore Sudanese refugee women’s narratives around parenting teenagers in the resettlement environment. | Narrative inquiry | Australia | - Sudanese  - Refugee | - 17 mothers and guardians  - Interviews |
| Lewig  2010 | To examine why recently arrived families from refugee backgrounds are presenting in the child protection system and to identify culturally appropriate strategies for intervention. | Qualitative descriptive | Australia | - Sudanese, Burundian/Congolese, Liberian, Somali, Iraqi, Iranian and Vietnamese  - Refugee | - 100 women and 30 men  - Focus groups |
| Liamputtong 2006 | To examine the lived experience of motherhood among Cambodian, Lao and Vietnamese immigrant women in Australia. | Ethnography | Australia | - Cambodian, Lao and Vietnamese  - Refugee | - 67 women  - Interviews |
| Lo  2016 | To examine mothers’ caretaking experiences across two settings: at schools and in health care facilities. | Grounded theory | United States | - Mexican  - Undocumented | - 25 mothers  - Interviews |
| Longman 2013 | To provide insight on how parenting can be conceived as a citizenship practice. | Ethnography | Belgium | - South American, African and Middle-Eastern  - Undocumented | - 6 mothers  - Interviews, observation |
| Losoncz  2016 | To describe the complexities of parenting in a new social, structural, cultural, and legal environment. | Qualitative descriptive | Australia | - South Sudanese  - Refugee | - 11 women and 21 men  - Interviews |
| Lunneblad 2012 | To examine interactions between parents and teachers and to see how these are colored by power, trust/mistrust and notions of "other". | Narrative inquiry | Sweden | - Arabic, Somali, and Turkish speaking  - Refugee | - 3 mothers, 10 fathers  - Focus groups |
| Lykes  2013 | To better understand, in parents’ own voices, their embrace of and resistance to direct communication with their children about the threat of deportation. | Narrative inquiry and qualitative descriptive ^[[7]](#footnote-7)^ | United States | - Latino  - Undocumented | - 18 parents (57% women and 43% men) from one study, 132 parents (70% women) from a second study and others who participated in workshops/  meetings (n=NR)  - Interviews, conversations in meetings/  workshops, and survey with open ended questions |
| Machado- Casas  2009 | To explore the ways in which parents transmit funds of knowledge to their undocumented youth to prepare them for adult life. | Narrative inquiry | United States | - Latino (Mexican, Guatemalan and Salvadorian)  - Undocumented | - 1 mother, 2 fathers  - Interviews |
| Mantovani 2014 | To explore the experience of discovery of pregnancy, attempts to seek professional help and the eventual decision to continue with the pregnancy. | Qualitative descriptive | United Kingdom | - Black African (South West Africa, West Africa and East Africa)  - Asylum-seeker | - 15 women  - Interviews |
| Marcelletti Rocha de Oliveira  2015 | To describe maternal migration has influenced care arrangements and education trajectories of children in new country (and of those who remained in home country). | Ethnography | United States | - Mexican  - Undocumented | - 20 mothers  - Interviews, observation and text messages |
| Martinez  2010 | To examine how people eat in ways to maintain their family’s overall well-being, and how what they eat is changing because of transnational, modernizing practices and discourses. | Ethnography | United States | - Latino  - Undocumented | - 15 mothers  - Interviews and observation |
| Maternowska 2014 | To focus on changing views of gender roles, masculinity and relationship dynamics in the context of migration in order to explain low levels of reproductive healthcare utilization (family planning, reproductive decision making). | Grounded theory | United States | - Mexican  - Undocumented | - 23 men  - Interviews |
| Matthiesen  2016 | To show how institutional and interactional processes in the parent–teacher conference systematically silence parents’ voices. | Case study | Denmark | - Somali  - Refugee | - 4 mothers, 1 father  - Interviews and observation |
| McMichael 2013 | To examine the ways young women with refugee backgrounds negotiate teen pregnancy and early motherhood. | Ethnography | Australia | - Migrants from Sudan, Ethiopia, Liberia, Uganda, Burundi, Iraq, Afghanistan, Iran, Kuwait, Bosnia, Croatia and Burma  - Refugee | - 8 mothers  - Interviews, informal discussions, journal responses and field- notes |
| McNee  2015 | To describe mothers’ academic aspirations for their children in the US. | Qualitative descriptive | United States | - Latinas (Guatemala, Nicaragua, El Salvador, and Colombia)  - Undocumented | - 4 mothers  - Interviews and focus groups |
| Medina  2008 | To understand how parents are using their cultural background, prior educational experiences in Mexico/Latin America to understand the educational system and how they access pre-college information. | Ethnography | United States | - Mexican  - Undocumented | - 6 mothers  - Interviews, observation and focus groups |
| Moya Salas  2008 | To explore the cultural values of first generation Mexican immigrant mothers and how they evolve with acculturation. | Grounded theory | United States | - Mexican  - Undocumented | - 12 mothers  - Focus groups and participation in activities (poetry writing, collage, photo voice) |
| Mwembo  2009 | To describe the educational experiences of Congolese immigrant families and their children attending K-12 schools. | Case study | United States | - Congolese  - Refugee | - 5 mothers, 5 fathers  - Interviews, observation and questionnaires |
| Nagasa  2014 | To elicit parents’ perspectives on their relationship for serving the educational needs of children. | Case study | United States | - Ethiopian  - Refugee | - 3 mothers, 1 father  - Interviews and field-notes |
| Napp-Avelli  2014 | To explore the knowledge and resources two Latino immigrant families have acquired through their experiences and how they use them to support their children’s education and mathematics education. | Case study | United States | - Latino  - Undocumented | - 2 mothers, 1 father, 1 aunt  - Interviews and observation |
| Nguyen  2012 | To examine the intersections of race, gender, class with language, cultural and technological barriers as reflected in the experience of parents in schools. | Ethnography | United States | - Vietnamese  - Refugee | - 16 mothers, 16 fathers  - Interviews |
| Nguyen  2013 | To investigate the parents’ perceptions of their roles, their perspectives and experiences as well as their strategies in maintaining cultural values in immigrant families. | Narrative inquiry | Canada | - Vietnamese  - Refugee | - 4 mothers, 4 fathers  - Interviews and field-notes |
| Nicol  2014 | To provide a deeper understanding of the refugee experience related to early oral health by exploring pre-school refugee families understanding of early childhood caries and child oral health, experiences of accessing dental services and barriers and enablers for achieving improved oral health. | Qualitative descriptive | Australia | - Migrants from Iraq, Kuwait, Burma, Sudan, Afghanistan, Burundi, DRC (Congo), Rwanda and Nigeria)  - Refugee | - 37 mothers, 2 fathers  - Focus groups |
| Nilsson  2012 | To describe parents' perceptions regarding their children’s adjustment. | Qualitative descriptive | United States | - Somali  - Refugee | - 24 women  - Focus groups |
| Ochala  2016 | To investigate the problems experienced by single African women of refugee background including challenges encountered in accessing services and the stigma of single parenthood within their cultures. | Qualitative descriptive | Australia | - African (Burundi, Sudan, Rwanda and Congo)  - Refugee | - 10 mothers  - Interviews |
| Osman  2016 | To explore refugees’ experiences and challenges of being parents in Sweden, and the support they  need in their parenting. | Qualitative descriptive | Sweden | - Somali  - Refugee | - 15 mothers, 8 fathers  - Focus groups |
| Peled  2013 | To shed light on the mothering experiences of sex-trafficked women. | Qualitative descriptive | Israel | - Migrants from the Former Soviet Union  - Undocumented (sex-trafficked) | - 8 women  - Interviews |
| Peregrine Antalis  2015 | To examine how refugee mothers negotiate the complex terrain of  mothering in the United States as they struggle to define themselves and citizens and subjects. | Ethnography | United States | - Migrants from Bhutan,  Burma, the Democratic Republic of Congo, Sudan and Iraq  - Refugee | - 20 mothers  - Interviews and observation |
| Perez  2010 | To explore what immigrant mothers perceived and expected from their children and the educational system prior to immigrating, as well as how these expectations and perceptions played a role in their child’s education during the acculturation process after immigrating. | Ethnography | United States | - Mexican  - Undocumented | - 8 mothers  - Interviews |
| Ramsay  2016 | To show how the child welfare system functions as an instrument to govern women to ‘fit’ with an idealized standard of citizenship in Australia. | Ethnography | Australia | - Black African (Congo, Rwanda and Burundi)  - Refugee and asylum-seeker | - 35 women  - Interviews and observation |
| Ramsden  2013 | To analyze perceptions of the education systems pre- and post-migration and implications of these for their family’s ability to adapt and cope with life in the new country. | Case study | Australia | - Somali  - Refugee | - 28 parents (11 mothers, 4 fathers, others NR)  - Interviews and focus groups |
| Rasmussen  2012 | To examine West African immigrants’ perceptions of child welfare authorities and the role of disciplining and monitoring in these communities’ meaning making. | Grounded theory | United States | - Migrants from West Africa (Sierra Leone, Liberia, Gambia, and Guinea)  - Refugee | - 19 mothers, 13 fathers  - Interviews and focus groups |
| Renzaho  2012 | To assess intergenerational differences in food, physical activity, and body size perceptions among refugees and migrants. | Grounded theory | Australia | - African (Sudanese, Somali, and Ethiopian)  - Refugee | - 25 women and 9 men  - Interviews and focus groups |
| Reyes  2014 | To understand the role of parental support in the educational success of their children. | Phenomenology | United States | - Mexican  - Undocumented | - 9 mothers, 9 fathers  - Interviews |
| Reyes Cruz  2006 | To collect narratives on the experiences of undocumented Mexican immigrant parents working to make schools responsive and accountable. | Ethnography | United States | - Mexican  - Undocumented | - 13 mothers, 1 father  - Interviews, observation and field- notes |
| Riggs  2015 | To identify the sociocultural influences on child oral health in refugee and migrant communities. | Qualitative descriptive | Australia | - Pakistani (Muslim), Assyrian Chaldean (Iraq, Christian), Iraq (Muslim), Lebanese (Muslim), Iraqi/Lebanese  - Refugee | - 115 mothers, grandmothers, caregivers  - Interviews and focus groups |
| Saavala  2010 | To investigate how women of refugee and migrant background view their deliberative capacities in making choices and how this combines with their self-representations as mothers, daughters and wives. | Narrative inquiry | Finland | - Kosovo Albanian and Russian  - Refugee | - 30 women  - Interviews |
| Sanchez  2013 | To focus on the mothering experiences of an undocumented mother of a Mexican working class family of mixed-legal status. | Case study | United States | - Mexican  - Undocumented | - 1 family (mother was focus)  - Interviews and observation |
| Schmalzbauer  2009 | To analyze the ways in which context of reception affects gender relations in terms of three primary elements of migrant incorporation: employment, geography and culture. | Ethnography | United States | - Mexican  - Undocumented | - 21 women and 12 men  - Interviews and observation |
| Shandy  2008 | To look at the experiences of pregnant and post-partal African women to explore questions surrounding use of maternity services and their relationship to larger issues of integration into Irish society. | Ethnography | North Ireland | - Migrants from Algeria, Angola, Cameroon, Côte d’Ivoire, Democratic Republic of the Congo, Liberia, Nigeria, Sudan, South Africa, Sierra Leon, Uganda, and Zimbabwe  - Asylum-seeker | - 51 women  - Interviews and observation |
| Shoaff  2010 | To describe the impact of the political, social and economic structures and power relations on the everyday experiences of Haitian women as workers, migrants, mothers, and activists. | Ethnography | Dominican Republic | - Haitian  - Undocumented | - Women (n= NR),  - Interviews and observation |
| Smith  2012 | To present information about the experiences of first generation West Indian/Caribbean families in New York City schools. | Grounded theory | United States | - West Indian/ Caribbean (Jamaica, Trinidad, and St Vincent)  - Undocumented | - 3 mothers, 1 father  - Interviews, observation, narrative account, historical research and memos |
| Speiglman  2013 | To analyze the factors that lead illegal and ineligible migrants to seek aid for their eligible children, the enrollment obstacles they face, and the ‘need assistance program’ ability to meet their needs. | Qualitative descriptive | United States | - Latino (mostly Mexican)  - Undocumented | - 32 participants (number of mothers and fathers NR)  - Focus groups |
| Stacciarini  2015 | To describe rural Latino immigrant mother and adolescent dyads’ perceptions of familial and community environments in their lives and how these contexts affect their mental well-being. | Mixed methods  Qualitative descriptive | United States | - Latino  - Undocumented | - 31 mothers  - Interviews |
| Stack  2009 | To explore the role of leisure among Afghan refugees who have recently immigrated- how leisure helps them in adapting to their immigration processes. | Phenomenology | Canada | - Afghan  - Refugee | - 7 women and 4 men  - Interviews |
| Stewart  2015 | To examine challenges faced by refugee  new parents from Africa in Canada. | Mixed methods  Qualitative descriptive | Canada | - Migrants from Zimbabwe and Sudan  - Refugee and asylum-seeker | - 43 mothers, 29 fathers  - Interviews |
| Stock  2012 | To describe the everyday lives of migrant women and their children and to describe how migration and immobility in transit countries are gendered and gendering experiences. | Ethnography | Morocco | - African  - Undocumented | - 19 women and 5 men  - Interviews, and observation |
| Su  2011 | To describe how Cambodian American parents provide educational resources to their adolescents. | Ethnography | United States | - Cambodian  - Refugee | - 9 mothers  - Interviews |
| Tachble  2011 | To explore the fathering perceptions and experiences of Ethiopian newcomer fathers. | Phenomenology | Canada | - Ethiopian  - Refugee | - 10 fathers  - Interviews |
| Teniente Valderas  2015 | To capture what mothers are using to counter mainstream, patriarchal structures that are serving as gatekeeping entities to higher education. | Ethnography | United States | - Mexican  - Undocumented | - 9 mothers  - Interviews, observation and focus groups |
| Tingvold, Hauff  2012 | To examine the resources that Vietnamese refugee parents use in raising their adolescent youth. | Phenomenology | Norway | - Vietnamese  - Refugee | - 18 parents (number of mothers and fathers NR)  - Interviews and focus groups |
| Tingvold, Middelthon  2012 | To explore the influence of extended family members upon a small sample of Vietnamese refugee parents and their adolescents while they undergo acculturation through their long-term resettlement process. | Phenomenology | Norway | - Vietnamese  - Refugee | - 18 parents (number of mothers and fathers NR)  - Interviews and focus groups |
| Uchechukwu Ogbu  2014 | To explore the experiences of parenting in ‘direct provision’ (i.e., the living environment provided for asylum-seekers by government). | Qualitative descriptive | North Ireland | - Migrants from Eastern Europe (Albania) and Africa (Liberia, Nigeria, Kenya, Uganda, Zimbabwe and Cameroon)  - Asylum- seeker | - 16 parents (number of mothers and fathers NR)  - Interviews and focus groups |
| Ueland Sherack  2008 | To describe one family's bilingual, bicultural, and binational literacy experience. | Case study | United States | - Mexico  - Undocumented | - 1 mother, 1 father, maternal grandparents, - Interviews and observation |
| Urrieta  2011 | To explore parents’ and grandparents’ perspectives about why going to Nocutzepo in Mexico was important for their children and grandchildren’s cultural identities, even when their children were truant from US schools. | Ethnography | United States | - Mexican  - Undocumented | - 10 mothers, 10 fathers, 4 grandmothers, 4 grandfathers  - Interviews and observation |
| Valdez  2013 | To explore the consequences of increasingly restrictive immigration policies on social capital among Mexican mothers with unauthorized immigrant status. | Qualitative descriptive | United States | - Mexican  - Undocumented | - 21 mothers  - Focus groups |
| Van Ee  2013 | To assess parents’ internal representation, or working model of relationship with their child. | Mixed methods  Grounded theory | Netherlands | - Migrants from Middle East, Africa, East Europe, Asia, and South America  - Asylum-seeker and Refugee | - 29 fathers  - Interviews |
| Van Korlaar  2015 | To explore the experiences and involvement in their child’s education of nine Somali refugee parents. | Case study | United States | - Somali  - Refugee | - 8 mothers, 1 father  - Interviews, observation, focus groups, field notes, journals, artefacts and documents |
| Vervliet  2014 | To listen to the feelings and experiences of unaccompanied refugee mothers—young mothers living in another country and separated from their parents. | Narrative inquiry | Belgium | - Unaccompanied minors from Angola, Guinea, Kosovo, Burundi, Cameroon, Congo, Kenya, Yugoslavia, Sierra Leone, Sudan, Brazil, and Ivory Coast  - Refugee | - 20 mothers  - Interviews |
| Vesely  2013 | To explore the experiences of mothers as they selected and secured early childhood care and education (ECCE) for their young children. | Grounded theory | United States | - African (Ethiopia, Ghana, Sudan, Egypt, Eritrea, Morocco, Somalia and Latin America (El Salvador, Mexico, Guatemala, Argentina, Dominican Republic, Ecuador)  - Undocumented and Refugee | - 40 mothers  - Interviews |
| Vesely  2015 | To examine the economic experiences of immigrant families, including the process of building economic security. | Grounded theory | United States | - Migrants from Latin America and Africa: Ethiopia, Ghana, Sudan, Egypt, Eritrea, Morocco, Somalia, El Salvador, Mexico, Guatemala, Argentina, Dominican Republic, and Ecuador  - Undocumented and Refugee | - 40 mothers  - Interviews and observation |
| Viramontez Anguianao  2012 | To explore how different ecological factors (familial, social, and educational realities), within and outside the family, affected the educational success of the children of undocumented families. | Ethnography | United States | - Latino  - Undocumented | - 63 parents (number of mothers and fathers NR)  - Interviews |
| Vue  2011 | To examine perspectives on food habits, acculturation, and health among Hmong women with young children in northern California. | Grounded theory | United States | - Hmong  - Refugee | - 15 mothers  - Interviews |
| Watts  2011 | To explore the perceptions and attitudes of prehospital pediatric care (911) among Spanish speaking parents and to identify barriers to use and possible areas for improvement. | Qualitative descriptive | United States | - Spanish-speaking (predominantly Mexican)  - Undocumented | - 44 women and 5 men  - Focus groups |
| Watts  2015 | To solicit lived experiences of African, Australian young refugee women who have experienced early motherhood. | Phenomenology | Australia | - Migrants from Ethiopia, Egypt, Uganda, Guinea, Kenya, Tanzania, Liberia and Sierra Leone  - Refugee | - 16 mothers  - Interviews |
| White  2014 | To evaluate the effect of Alabama’s2011 omnibus immigration law on Latina immigrants and their US- and foreign-born children’s access to and use of health services. | Qualitative descriptive | United States | - Latino (mostly Mexico)  - Undocumented | - 30 mothers  - Interviews |
| Willen  2014 | To describe how illegality penetrates virtually every sphere of one Filipina migrant women, and how she crafts ‘inhabitable spaces of welcome’ in which how own existential imperatives and moral commitments are sustained despite the daily abjection she confronts. | Ethnography | Israel | - Filipina  - Undocumented | - 1 mother  - Interviews |
| Williams  2008 | To examine the overarching themes regarding cultural beliefs and values held by refugee participants on the parenting process and to understand why parents present in the child welfare system. | Qualitative descriptive | Australia | - Migrants from Sudan, Congo, Ethiopia, Liberia, Sierra Leone, Somalia, Sudan, Afghanistan and Iraq  - Refugee | - 24-45 participants (number of men and women NR)  - Focus groups |
| Xu  2012 | To describe how undocumented immigrants learn about services and navigate systems to access services for their citizen children and undocumented immigrants' experiences and barriers they face in accessing services for their citizen children. | Mixed methods  Qualitative descriptive | United States | - Latino  - Undocumented | - 12 mothers, 9 fathers  - Interviews and survey |
| Zannettino  2012 | To explore the factors that have an impact on domestic violence in African refugee communities. | Qualitative descriptive | Australia | - Liberian  - Refugee | - 30-40 women  - Focus groups |

1. The methodology was based on what was stated in the article. In cases where it was not explicitly named or it was unclear, we applied a label based on the description provided. General ‘qualitative’ exploratory studies were categorized as ‘qualitative descriptive’. ‘Interpretive description’ was also categorized as qualitative descriptive. For mixed methods research designs the qualitative component of the design was also recorded. [↑](#footnote-ref-1)
2. Migrant description was based on description (ethnicity and/or country of origin) in the study. Migrant group studied may have included other migrants, but only refugees, asylum-seekers and undocumented migrants highlighted in table. [↑](#footnote-ref-2)
3. Sample = parents (mothers, fathers, guardians and/or extended family members); if there was a mix of parents and others and the number of parents could not be identified other descriptors (e.g., families, women, men) were used. [↑](#footnote-ref-3)
4. Methods were categorized into: interviews (included semi-structured, informal, structured), observation (participant or non-participant observation), focus groups and other (e.g., photovoice, journals, field-notes, questionnaires, text review) [↑](#footnote-ref-4)
5. NR = not reported [↑](#footnote-ref-5)
6. This study combined data from two studies. [↑](#footnote-ref-6)
7. The article synthesized data from three studies. [↑](#footnote-ref-7)
